# Explainable AI in Diagnostic Radiology for Neurological Disorders: A Systematic Review, and What Doctors Think About It

**DOI:** 10.3390/diagnostics15020168

**Published:** 2025-01-13

**Authors:** Yasir Hafeez, Khuhed Memon, Maged S. AL-Quraishi, Norashikin Yahya, Sami Elferik, Syed Saad Azhar Ali

**Affiliations:** 1 Faculty of Science and Engineering, University of Nottingham, Jalan Broga, Semenyih 43500, Selangor Darul Ehsan, Malaysia; yasir.hafeez@nottingham.edu.my; 2 Centre for Intelligent Signal and Imaging Research, Department of Electrical and Electronic Engineering, Universiti Teknologi PETRONAS, Seri Iskandar 32610, Perak Darul Ridzuan, Malaysia; khuhed_22000210@utp.edu.my (K.M.); norashikin_yahya@utp.edu.my (N.Y.); 3 Interdisciplinary Research Center for Smart Mobility and Logistics, King Fahd University of Petroleum and Minerals, Dhahran 31261, Saudi Arabia; maged.quraishi@kfupm.edu.sa (M.S.A.-Q.); selferik@kfupm.edu.sa (S.E.); 4 Aerospace Engineering Department and Interdisciplinary Research Center for Smart Mobility and Logistics, and Interdisciplinary Research Center Aviation and Space Exploration, King Fahd University of Petroleum and Minerals, Dhahran 31261, Saudi Arabia

**Keywords:** brain MRI, neurological disorders, computer aided diagnosis, explainable artificial intelligence, deep learning, medical image analysis

## Abstract

**Background:** Artificial intelligence (AI) has recently made unprecedented contributions in every walk of life, but it has not been able to work its way into diagnostic medicine and standard clinical practice yet. Although data scientists, researchers, and medical experts have been working in the direction of designing and developing computer aided diagnosis (CAD) tools to serve as assistants to doctors, their large-scale adoption and integration into the healthcare system still seems far-fetched. Diagnostic radiology is no exception. Imagining techniques like magnetic resonance imaging (MRI), computed tomography (CT), and positron emission tomography (PET) scans have been widely and very effectively employed by radiologists and neurologists for the differential diagnoses of neurological disorders for decades, yet no AI-powered systems to analyze such scans have been incorporated into the standard operating procedures of healthcare systems. Why? It is absolutely understandable that in diagnostic medicine, precious human lives are on the line, and hence there is no room even for the tiniest of mistakes. Nevertheless, with the advent of explainable artificial intelligence (XAI), the old-school black boxes of deep learning (DL) systems have been unraveled. Would XAI be the turning point for medical experts to finally embrace AI in diagnostic radiology? This review is a humble endeavor to find the answers to these questions. **Methods:** In this review, we present the journey and contributions of AI in developing systems to recognize, preprocess, and analyze brain MRI scans for differential diagnoses of various neurological disorders, with special emphasis on CAD systems embedded with explainability. A comprehensive review of the literature from 2017 to 2024 was conducted using host databases. We also present medical domain experts’ opinions and summarize the challenges up ahead that need to be addressed in order to fully exploit the tremendous potential of XAI in its application to medical diagnostics and serve humanity. **Results:** Forty-seven studies were summarized and tabulated with information about the XAI technology and datasets employed, along with performance accuracies. The strengths and weaknesses of the studies have also been discussed. In addition, the opinions of seven medical experts from around the world have been presented to guide engineers and data scientists in developing such CAD tools. **Conclusions:** Current CAD research was observed to be focused on the enhancement of the performance accuracies of the DL regimens, with less attention being paid to the authenticity and usefulness of explanations. A shortage of ground truth data for explainability was also observed. Visual explanation methods were found to dominate; however, they might not be enough, and more thorough and human professor-like explanations would be required to build the trust of healthcare professionals. Special attention to these factors along with the legal, ethical, safety, and security issues can bridge the current gap between XAI and routine clinical practice.

## 1. Introduction

### 1.1. Neurological Disorders—Morbidity and Mortality

According to World Health Organization (WHO), neurological disorders are among the top three killers in the world, even in developed countries with adequate healthcare infrastructure [1]. Neurodegenerative disorders like Alzheimer’s disease (AD) and Parkinson’s disease (PD) can drastically degrade quality of life. Unlike other parts of the body, brain cells do not regenerate, and hence early diagnosis is of paramount significance to contain the progression of such diseases [2,3,4,5,6]. However, at the early stage of such disorders, diagnosis is challenging [2] due to very subtle changes in medical imaging data. In addition, some diseases in their infancy may present similar or overlapping findings, especially in cases of neurodegenerative disorders, thereby rendering the differential diagnosis even more tedious [7,8,9]. This would require highly skilled and experienced medical professionals for accurate diagnoses, choosing the right course of treatment, and containing the prognoses. The unavailability of such experts and the lack of infrastructure, especially in under-developed countries, can produce catastrophic outcomes.

### 1.2. AI in CAD

AI has revolutionized every walk to life, and healthcare and medicine are no exceptions [6,10,11]. Over the past couple of decades, tremendous research has been observed for the design and development of CAD tools for multimodal data to act as assistants to domain experts in reaching fast and concrete diagnoses. From old-school computer vision (CV) algorithms, to machine learning (ML), to the more recent DL architectures, great progress has been observed in this field, producing outstanding results [6,10,12,13,14,15]. Radiological imaging has become an inseparable part of the diagnosis process, with technologies like X-ray, ultrasound, MRI, CT, and PET scans playing a vital role in assisting experts in accurate diagnoses. MRI, being a non-invasive and highly informative modality, is found to be widely employed [13,16]. DL-powered machines have the ability to look into the most intricate features, even at pixel level, which the human eye might overlook. In this domain, convolutional neural networks (CNNs) have recently shown unprecedented promise [10,17,18,19] and have been employed in systems with multiple disease diagnosis capabilities. Some researchers have even observed that the performance of such DL-powered systems is comparable to humans in real-world tasks, and in some cases might surpass human domain experts’ performance in terms of speed and accuracy [10,19,20], but this increased performance and accuracy of DL-powered systems comes at a price.

Before taking the discussion any further, we would like to take a moment here to introduce some standard terminologies pertaining to XAI:

1. Interpretability: Interpretability refers to the ability to understand the decision-making process of an AI model. The operation of an interpretable model is transparent and provides details about the relationships between inputs and outputs.
2. Explainability: Explainability refers to the ability of an AI model to provide clear and intuitive explanations of the decisions made to the end user. In other words, an explainable AI model provides justification of the decisions made.
3. Transparency: Transparency refers to the ability of an AI model to provide a view into the inner workings of the system, from inputs to inferences.
4. Black box: The black box model in AI is one whose operations are not visible to the user. Such models arrive at decisions without providing any explanation as to how they were reached. Such models lack transparency, and are therefore frowned upon and not trusted in applications like diagnostic medicine, where precious human lives are on the line.

**Figure 1:**
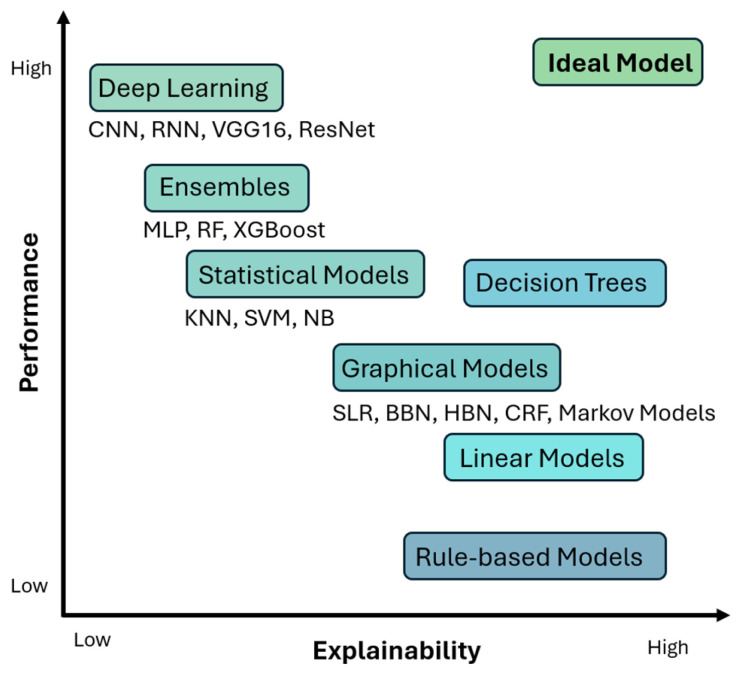
Accuracy/interpretability trade-off.

The journey with AI started with simpler rule-based algorithms in ML, like decision trees, which provided clear rules for end users to understand the reasons for the classifications. The features in ML-powered systems were hand-crafted by developers [16] and thus offered higher levels of transparency in their inferences and decisions. The accuracy of such systems was relatively low. In order to increase accuracy and performance, complex DL architectures were developed with many hidden layers and millions of trainable parameters. The features in such systems were extracted implicitly and hence the opacity increased drastically [21]. Although there were leaps in accuracy, the decision-making process was wrapped in a “black box” [6,10,11,15,20,22,23,24,25,26,27], resulting in a decrease in trust, especially in highly sensitive fields like diagnostic medicine, where a single wrong decision can be a matter of life and death [6,19,27,28,29,30]. This trade-off between accuracy and explainability [6,14,15,31] in the evolution of AI is shown in Figure 1. This has been one of the leading factors behind the fact that, despite the tremendous performance peaks achieved by such DL-powered CAD tools, they have not yet been able to find their way into routine medical practices, since both doctors and patients, by all means, demand their right to know the reasons for a particular diagnosis/inference generated by such CAD tools [19,28]. This gave rise to a recent sub-domain of AI called XAI.

### 1.3. Unraveling the Mystery!

**Figure 2:**
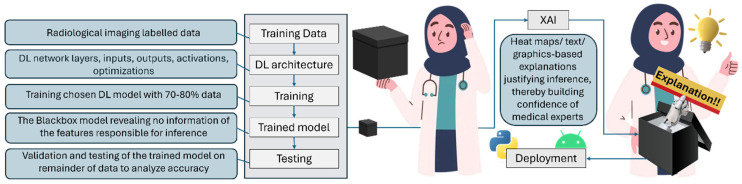
Block diagram of the journey from the old-school black box models to XAI.

XAI is an attempt by engineers to demystify the otherwise secretive working of complex DL architectures popularly referred to as “black boxes”, which is the leading cause of mistrust for people [28,32]. Figure 2 shows a block diagram of the end-to-end working of an XAI-powered DL regimen (from training to deployment), along with the tentative expressions and comfort level of domain experts at various stages.

Many theories, methods, and frameworks have been devised to provide plausible explanations for the outcomes of such models.

### 1.4. XAI Methods and Frameworks

XAI methods have been classified into various categories and many respective frameworks have been developed over a brief period of time [28], but explaining these in detail is beyond the purview of this work; hence, we will state them succinctly.

Generated explanations can be textual, numerical, visual or example-based. In the case of radiological imaging, most researchers have worked with visual explanations in the form of heatmaps, which highlight the regions in the input images that contribute to a particular inference [25]. From the point of view of scope, the explainability of a model can either be local or global: a global explanation explains the behavior of an entire model based on the entire input dataset, whereas a local explanation might just use a couple of examples to help explain why certain decisions were made. From the point of view of the implementation stage, XAI methods can be categorized as ante-hoc and post-hoc depending on whether the explanations were generated during or after training. In terms of the applicability of XAI methods, we have model-specific and model-agnostic approaches. Model-agnostic approaches are generally easy to implement, since their applicability is independent of the underlying AI model, and are also sometimes referred to as plug-and-play [16]. The remaining part of this section briefly introduces the most commonly employed XAI frameworks.

**Figure 3:**
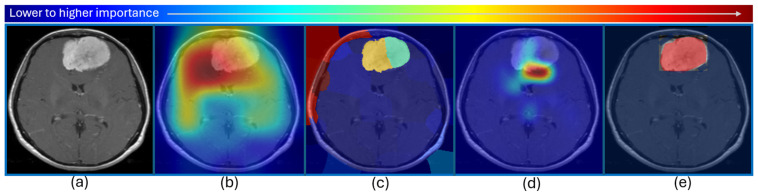
Brain MRI with tumor taken from the Kaggle dataset [35], correctly classified by MobileNetV2, which was trained with 154 images with a tumor (class 1) and 97 images without a tumor (class 2) for this demonstration. The figure shows (**a**) raw MRI, and heatmaps to highlight regions responsible for classification generated by (**b**) Grad-CAM, (**c**) LIME, and (**d**) OSA using MATLAB R2024b. A ‘jet’ color scheme used to highlight the image based on the influence of different regions leading to this inference (Tumor) by the DL model. The ‘jet’ color map has deep blue as the lowest value and deep red as the highest, as shown at the top of the figure. Notice the inaccuracies of the heatmaps in (**b**–**d**), highlighting irrelevant regions, as shown in (**b**), and missing critical tumorous regions, as shown in (**d**). This is primarily due to the primitive nature of the dataset employed to train the DL regimen used here for demonstration purposes and can be improved further in practical scenarios. An ideal heatmap (generated manually) is shown in (**e**), where only the tumor region appears as “most significant” (red), and all other pixels appear as “least significant” (blue) for this brain tumor classification example.

Local Interpretable Model-Agnostic Explanations (LIME) [33] generates explanations by perturbing the input data of a model and observing the changes in the output. It can thus highlight the significant features of input data responsible for a particular decision. SHapley Additive exPlanations (SHAP) assigns weights to all input features and observes the outcomes of all weighted input feature combinations. Gradient-weighted class activation mapping (Grad-CAM) [34] works with CNNs to identify important regions in an input image which are responsible for the inference. It is applied using gradient information from the output layer to produce a heatmap for the input image. Layer-wise relevance propagation (LRP) also generates similar heatmaps by assigning relevance scores to all neurons in the output layer of a CNN, and then backpropagates to the input layer, while computing scores for every neuron. Occlusion sensitivity analysis (OSA) and saliency maps (SM) are frequently used schemes to generate visual explanations for image input data. Their mode of operation is similar. In OSA, patches of images are occluded periodically and the corresponding effects on the outputs are observed. If the probability of a certain prediction drops drastically by occluding a certain input image patch, it would signify that the patch holds important information for that particular prediction. SM also generates heatmaps in a similar manner. The difference is that, in case of SMs, each pixel from the input is removed iteratively and the corresponding drop in probability of inference defines its importance. Hence, the heatmap generated by SM contains all the significant pixels responsible for a certain prediction. In the case of a CAD tool for neurological disorders, for example, all brain MRI regions responsible for the diagnosis of a certain disease would light up for experts to visualize the reason/explanation for that particular diagnosis by the tool. In order to elaborate the visual explanations of XAI frameworks for clear understanding, a simple brain tumor dataset was downloaded from Kaggle [35], and a two-class DL model was trained to classify MRI images as ‘Tumor’ or ‘No tumors’. The visual explanations generated by different XAI techniques are shown in Figure 3.

### 1.5. Would XAI Be the Matchmaker?

The next important questions are:

“Would the integration of explainability to the otherwise opaque DL architectures (rejected by doctors and patients [36]) bridge the gap and develop the trust of domain experts in using CAD tools?”

“Would only visual explanations be enough for experts in diagnostic radiology?”

“What else should be done to pave the way for the large scale incorporation of AI into diagnostic medicine?”

With the help of the literature and medical experts, we will be looking for answers to these and other such questions in this humble endeavor.

The rest of this paper is organized in such a way that methods are given in Section 2, and the the main findings for the systematic review, prior art pertaining to brain MRI-based CAD of neurological disorders using both AI and XAI and medical domain experts’ opinions about this technology are presented in Section 3. Section 4 presents the discussion and the challenges ahead, and the paper is concluded in Section 5, which presents the answers we seek.

## 2. Methods

This section presents details pertaining to the study selection, inclusion/exclusion criteria, as well as the highlights, information extracted from the literature, and the statistics about article sources, neurological disorders studied, and XAI techniques and datasets employed in XAI-powered CAD research.

### 2.1. Study Selection

**Figure 4:**
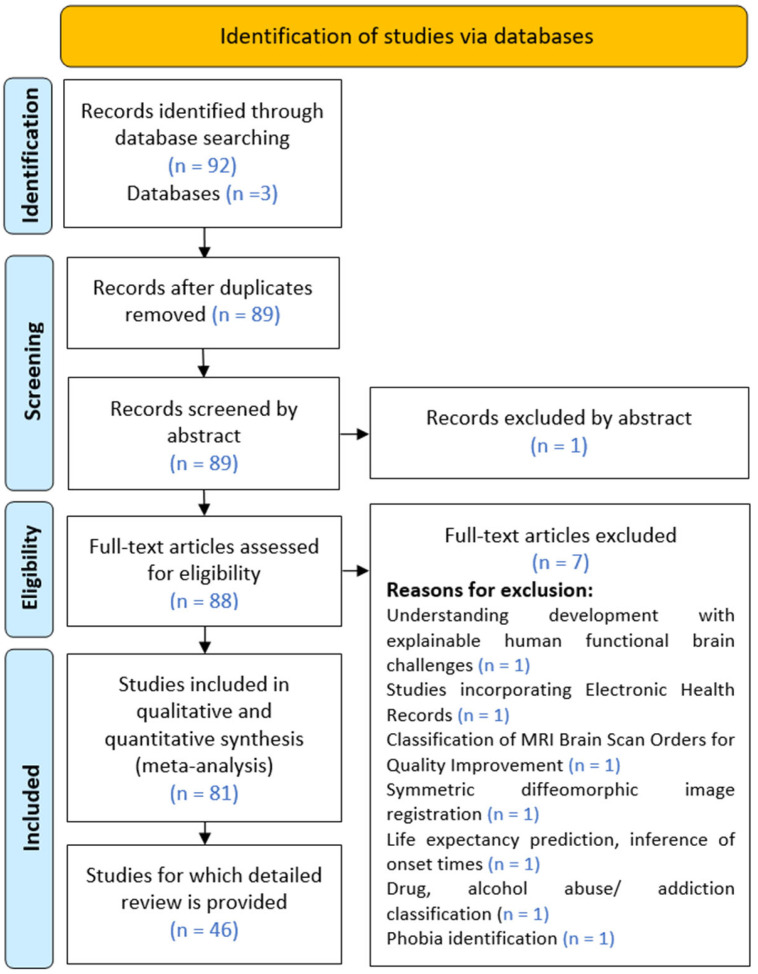
PRISMA study selection diagram: out of the 92 articles identified, 81 were included in the qualitative analysis.

The host databases Scopus, Web of Science, and Google Scholar underwent keyword searches for articles published up to November 2024. The search phrases used include “explainable AI in brain MRI-based computer-aided diagnoses”. Research conducted was scanned collectively from 2017 to 2023, while for the year 2024, it was scanned individually using filters. The titles of articles included in first 10 pages were examined and the most relevant were downloaded; only those with full-text access were downloaded. After the removal of duplicates, these articles were later screened by abstract. This was followed by a thorough examination of the full texts. The Preferred Reporting Items for Systematic Reviews and Meta-Analyses (PRISMA) [37] guidelines were followed in the process, as shown in Figure 4.

### 2.2. Inclusion Criteria

Research related to XAI-powered CAD of neurological disorders using ML and DL with MRI as the sole data modality was primarily included in this review. A few multimodal studies were also included. The CAD of neurodegenerative disorders was also included, along with that of neoplasms. Models that handle multiple types of MRI data, including different structural variants, were also considered in this review. Review articles were also included in the qualitative synthesis.

### 2.3. Exclusion Criteria

Various articles were excluded from this review based on certain criteria, which are enumerated here:

1. Understanding development with explainable human functional brain challenges
2. Studies incorporating electronic health records
3. Classification of MRI brain scan orders for quality improvement
4. Symmetric diffeomorphic image registration
5. Life expectancy prediction, inference of onset times
6. Drug, alcohol abuse/addiction classification
7. Phobia identification

### 2.4. Highlights and Information Extracted

The following information was extracted and summarized from the articles reviewed in this study:

1. Year of study
2. Diseases researched
3. Modalities employed
4. AI techniques used
5. Accuracy of developed systems
6. Algorithms used for Explainability
7. Datasets used

**Figure 5:**
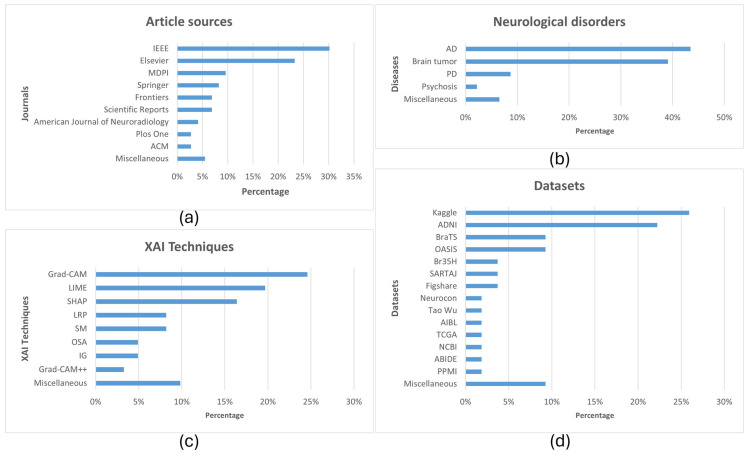
Statistics: (**a**) article sources—IEEE dominant; (**b**) neurological disorders—diagnosis for AD and brain tumors dominant since they are the most widely researched disorders in CAD research; (**c**) XAI techniques—Grad-CAM, LIME, and SHAP dominant; (**d**) datasets—ADNI (for AD and its sub-types) dominant alongside Kaggle. The percentages have been computed from a total of 81 articles.

Most articles were selected from IEEE, followed by Elsevier publications, as shown in Figure 5a. AD and brain tumor were the top two diseases researched (Figure 5b). The top two classification techniques applied were CNNs and transfer learning (TL). From the point of view of explainability, Grad-CAM and LIME were the top two employed techniques, closely followed by SHAP (Figure 5c). Kaggle was found to be the most used platform for dataset sharing, in addition to the Alzheimer’s Disease Neuroimaging Initiative (ADNI) dataset, which was the second most commonly used (Figure 5d).

## 3. Results

This section presents the key findings of the systematic review and sheds light on the applications of AI and XAI in the CAD of neurological disorders. It consolidates the research encompassing approaches including ML, CNNs, and TL, among others, in addition to presenting findings from research catering to multimodal data, the evaluation of clinical XAI, and medical experts’ feedback. Furthermore, we present opinions, concerns, and expectations from medical domain experts across the globe about XAI-powered CAD tools as their assistants in routine healthcare practice.

AI and XAI in CAD of Neurological Disorders

Despite the nearly negligible penetration of AI in current routine healthcare regimens (due to its multitude of limitations and unreliability), the massive amount of recent research, progress, and development in CAD from a data science perspective is sincerely praiseworthy. This section highlights the marvels achieved by this technology in diagnostic medicine (radiology, to be specific), in its journey from black box AI to the more recent and transparent XAI, for CAD of neurological disorders.

### 3.1. AI Applications in CAD

Numerous systems have been developed, ranging from recognizing MRI sequences and view planes [38] to preprocessing [39], segmentation of brain regions or anomalies [40] like tumors [41], and diagnosing disorders from a given MRI in multi-class problems [42]. This has only been possible mainly due to a couple of factors. The most important among them is the availability of massive, open access, publicly available, labeled datasets for data scientists and engineers to develop and train complex frameworks, rendering them capable of producing accurate inferences from unseen data in real-time. In addition, the very recent boost in the storage and processing capabilities of our machines to perform rapid calculations with millions of trainable parameters in deep and complex models has been a game changer for this technology [20,29]. Hats off to the drastic evolution of graphics processing units (GPUs) that made this possible. DL is known to be data-hungry [11]. That is, it can produce better results if trained on massive amounts of annotated/labeled data [43]. In case of brain MRI, the annotations/labels coming from experts (consultant radiologists/neurologists) are considered to be the “Gold Standard”. However, this can become an extremely time-consuming and tedious job [44] given the magnitude of data available online [45]. Moreover, incorrectly labeled data can obviously lead to poor training, which in turn results in poor accuracy of the models. Some pathologies are better visualized in specific MRI sequences as compared to others. For example, demyelinating diseases like multiple sclerosis (MS) and neuromyelitis optica (NMO) produce plaques/lesions on the brain, which are prominent as hyper-intense regions on a FLAIR (fluid-attenuated inversion recovery) sequence [45,46]. Therefore, a study on the computer-aided differential diagnosis (CADD) of MS and NMO should technically focus on FLAIR MRI. To assist such applications, various systems have been developed to automatically identify the sequence and view planes of MRI scans [47,48,49,50,51]. In addition, extra-cranial tissues, including the skull, eyes, neck, etc., can be a source of noise for an AI system being designed to assist in differential diagnosis of diseases in the brain. To help in such scenarios, various brain extraction tools (skull-strippers) have been developed with extraordinary (radiologist-like) capabilities to handle all MRI sequences and orientations. Such preprocessing techniques (Synthstrip [39], NeuroImaging Volumetric Extractor—NIVE [52]) have been found to increase the accuracy of CAD tools. Two main problems that AI is generally found to be working on are segmentation and classification. From the literature, AD and brain tumors were the most widely researched disorders in developing AI-powered CAD tools using brain MRIs as inputs. DL architectures have been widely used to segment tumors and lesions from MRI scans. Analysis of the texture and morphology of such tumors and lesions can further lead to accurate diagnoses. For example, the lesions appearing in MS and NMO on the brain can appear very similar [53]. A concrete differential diagnosis from this modality alone can thus be extremely challenging [54,55,56], resulting in delays incurred due to additional testing. AI can assist in reducing such overheads of cost and time. Two-dimensional and three-dimensional DL architectures, especially CNNs, have been found to be widely employed in multiple CAD systems [57]. Systems have been found that claim to classify as many as 35 diseases [58]. The major issue in such systems reported in the literature is their generalizability. That is, although some of these systems have been reported to have an accuracy as high as 100% in classifying multiple diseases and their sub-types [59,60,61,62,63,64], these systems fail to be as accurate when tested on unseen data from different sources and not used in training. In addition, the research discussed thus far has no embedded explainability, which means that the end user (doctor) has no idea of what is going on within the DL model and what the reason was for a particular diagnosis. The opacity of such systems is also one of the leading reasons for healthcare professionals’ mistrust of AI systems, and thus demands massive attention. From this point onwards, this section discusses XAI-powered CAD tools as an attempt by researchers and data scientists to bridge the gap between AI and healthcare by making CAD systems transparent, thereby building the confidence of medical experts in these assistive tools.

### 3.2. XAI Research in CAD

XAI has very recently emerged as a sub-domain of AI to assist domain experts in diagnoses and prognoses. From the statistical point of view of incorporating explainability into CAD systems using medical imaging data, X-rays have been widely researched, closely followed by MRI [27]. Among MRI studies, structural/anatomical MRI have been widely used, followed by functional MRI [16]. The chest and brain are the top two most researched anatomical locations [16], followed by the eyes and breasts [27]. From the point of view of explanations, over a period from 2017 to 2020, visual explanations were employed the most, with a constantly increasing trend, followed by textual and example-based explanations [27]. In the domain of visual explanations, perturbation methods have been found to dominate XAI algorithms [10]. Among XAI techniques, CAM and Grad-CAM are found to be the leading techniques, followed by LRP, guided backpropagation, LIME, and SHAP, among others [16]. In XAI-powered CAD research, AD and brain tumors are widely researched, followed by PD and others.

**Figure 6:**
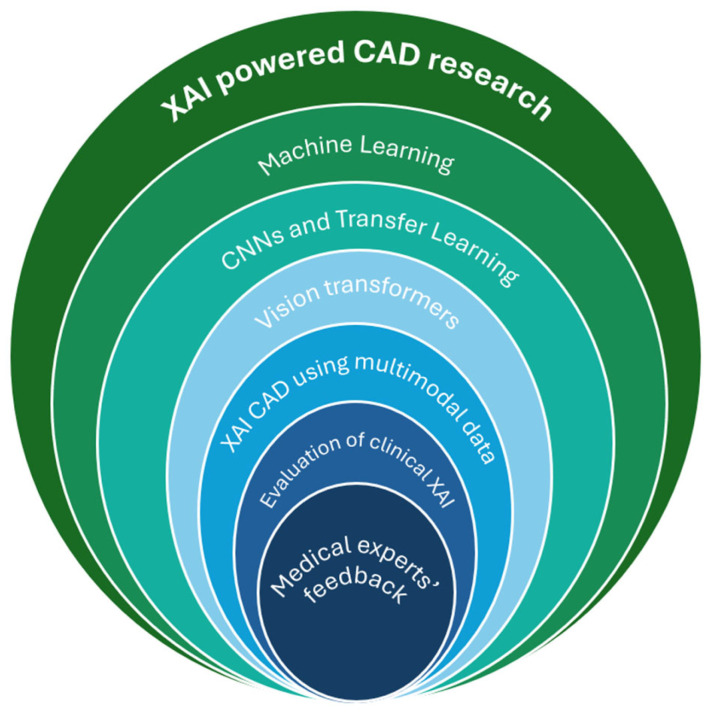
XAI-powered CAD research discussed in this study.

The rest of this section describes research on XAI-powered CAD in detail and has been categorized into various subsections, as depicted in Figure 6.

#### 3.2.1. Machine Learning

ML is known to extract knowledge from data using simpler methods like decision trees and linear regression. DL, on the other hand, is more complicated and relies on advanced methods like artificial neural networks. ML requires much less data and its operations are transparent, which is not the case in DL. ML might require human intervention in terms of feature engineering, whereas DL has the capabilities to reach predictions on its own after training on data, which, in most cases, requires high-end machines using GPUs. Most of the research on XAI-powered CAD has been found to employ DL and TL, which are presented in the subsequent section. This section summarizes the scant research from the literature on ML in Table 1.

#### 3.2.2. CNNs and Transfer Learning

A CNN is a type of DL neural network architecture used in applications such as CV, with the capabilities to extract features from input data. The network architecture consists of various layers, including the input layer (where the image input is applied), convolutional layers (which apply filters for feature extraction), pooling layers (which down-sample data to reduce computations), and fully connected layers (which make the final predictions). Despite the opacity of the operations of such architectures, they have been widely employed in medical imaging applications. TL is another important derivative that has been found to be immensely powerful. In this technique, the knowledge acquired by the network, when it is trained on one dataset for a particular task, can be used to improve model performance in another similar task. In other words, TL uses what has been learned in one setting to improve generalization in another setting. This technique can save time and resources because it reuses knowledge by fine-tuning the model instead of training a new model from scratch. A lot of research on CAD has been observed in the literature to employ DL and TL and is summarized in Table 2.

The study in [3] employs CNN and LIME for the diagnosis of AD. The system has been trained using the ADNI dataset and claims to have a classification accuracy of 94.96%. Many similar studies [4,5,18,66,67,68,69,70,71,72] were found to use different DL and XAI architectures for the diagnosis of AD, with some handling its sub-types as well. The common problem with these systems was relying on the ADNI dataset only for training and testing their systems. In practical scenarios, such systems are bound to suffer from a drastic reduction in accuracy due to poor generalizability. Moreover, they fail to provide any concrete quantitative or qualitative analysis of the explanations generated by their systems. Additionally, no doctors (domain experts and the ultimate end users) were found to be onboard with using these systems for evaluations. Similar issues and limitations were found in brain tumor research [4,5,18,66,67,68,69,70,71,72]. In [2], Camacho et al. present their work on explainable classifications of PD. They use a large multi-center database of T1-weighted (T1w) MRIs to train their CNN model with saliency maps to identify the regions responsible for inferences. Employment of data from about 14 centers caters to the generalizability issue, but the other questions pertaining to explainability still remain unanswered.

**Table 2:** XAI CAD research for neurological disorders using CNNs and TL techniques. The table contains the year of study, the pathology diagnosed, the modality used, the AI technology employed, the accuracy of the proposed system, the XAI technology embedded, and the dataset used for training the systems.

Study	Pathology	Modality	Technology	Accuracy	XAI	Dataset
[73] 2024	PD	MRI T1w	12 pre-trained CNN models	VGG19 best performance	Grad-CAM	PPMI (213 PD, 213 Normal Control (NC)), NEUROCRON (27 PD, 16 NC) and Tao Wu (18 PD, 18 NC)
[74] 2024	AD, progressive Mild Cognitive Impairment (pMCI), stable MCI (sMCI)	MRI	2D-CNN, TL	AD-CN 86.5%, sMCI-pMCI 72.5%	3D attention map	ADNI (AD 191, pMCI 121, sMCI 110, NC 204 subjects)
[75] 2024	Very mild dementia, moderate dementia, mild dementia, non demented	MRI	DenseNet121, MobileNetV2	MobileNetV2 93%, DenseNet121 88%	LIME	OASIS
[76] 2024	Brain tumor	MRI	Disease and spatial attention model (DaSAM)	Up to 99%	-	Figshare and Kaggle datasets
[77] 2024	Brain tumor	MRI	VGG16	99.4%	Grad-CAM	Kaggle and BraTS 2021 dataset
[78] 2024	Brain tumor	MRI FLAIR, T1, T2w	CNN	98.97%	-	3300 images from BraTS dataset
[79] 2024	AD	MRI	Ensemble-1 (VGG16 and VGG19) and Ensemble-2 (DenseNet169 and DenseNet201)	up to 96%	Saliency maps and Grad-CAM	Kaggle and OASIS-2 (896 MRIs for mild dementia, 64 moderate dementia, 3200 non-dementia, and 2240 very mild dementia)
[80] 2024	Glioma, Meningioma, Pituitary tumor	MRI	CNN	80%	LIME, SHAP, Integrated Gradients (IG), and Grad-CAM	7043 images from Figshare, SARTAJ, Br35H datasets
[81] 2024	AD	MRI	CNN	Real MRI 88.98%, Real + Synthetic MRIs 97.50%	Grad-CAM	Kaggle—896 MRIs for Mild Impairment, 64 Moderate Impairment, 3200 No Impairment, 2240 Very Mild Impairment. Synthetic images generated using Wasserstein Generative Adversarial Network with Gradient Penalty (WGAN-GP)
[82] 2024	Brain tumor	MRI T1w	10 TL frameworks	Up to 98% for EfficientNetB0	Grad-CAM, Grad-CAM++, IG, and Saliency Mapping	Kaggle—926 MRI images of glioma tumors, 500 with no tumors, 901 pituitary tumors, and 937 meningioma tumors
[83] 2024	Brain tumor	MRI	ResNet50	98.52%	Grad-CAM	Kaggle
[84] 2024	AD, MCI	MRI	CNNs with a multi-feature kernel supervised within-class-similar discriminative dictionary learning (MKSCDDL)	98.27%	Saliency maps, Grad-CAM, Score-CAM, Grad-CAM++	ADNI
[85] 2024	Brain tumor	MRI	Physics-informed deep learning (PIDL)	96%	LIME, Grad-CAM	Kaggle—glioma 1621 images, meningioma 1645, pituitary tumors 1775, and non-tumorous scans 2000 images
[86] 2024	Brain tumors four classes: glioma, meningioma, no tumor, and pituitary tumors	MRI	VGG19 with inverted pyramid pooling module (iPPM)	99.3%	LIME	Kaggle—7023 images
[2] 2023	PD	MRI T1w	CNN	79.3%	Saliency maps	1024 PD patients and 1017 age and sex matched HC from 13 different studies
[87] 2023	Brain tumor	MRI	VGG16	97.33%	LRP	1500 normal brain MRI images and 1500 tumor brain MRI images—Kaggle
[3] 2023	Non-dementia, very mild, mild, and moderate	MRI	CNN	94.96%.	LIME	ADNI
[72] 2023	AD, MCI	DW-MRI	CNN	78% for NC-MCI (45 test samples), 91% for NC-AD (45 test samples) and 81% MCI-AD (49 test samples)	Saliency map visualization	ADNI2 and ADNI-Go—152 NC, 181 MCI and 147 AD
[18] 2023	AD	MRI	3D CNN	87%	Genetic algorithm-based Occlusion Map method with a set of Backpropagation-based explainability methods	ADNI—145 samples (74 AD and 71 HC)
[88] 2023	Brain tumor	MRI	VGG16, InceptionV3, VGG19, ResNet50, InceptionResNetV2, Xception, and IVX16	95.11%, 93.88%, 94.19%, 93.88%, 93.58%, 94.5%, and 96.94% for VGG16, InceptionV3, VGG19, ResNet50, InceptionResNetV2, Xception, and IVX16, respectively	LIME	Kaggle—3264 images
[12] 2022	Brain tumor (classification and segmentation)	MRI	ResNet50 for classification, encoder–decoder neural network for segmentation	-	Vanilla gradient, guided backpropagation, integrated gradients, guided integrated gradients, SmoothGrad, Grad-CAM, and guided Grad-CAM visualizations	BraTS challenges 2019 (259 cases of HGG and 76 cases of LGG) and 2021 (1251 MRI images with ground truth annotations)
[89] 2022	Brain tumors (meningioma, glioma, and pituitary)	MRI	CNN	94.64%	LIME, SHAP	2870 images from Kaggle
[67] 2022	Early-stage AD dementia	MRI	EfficientNet-B0	AUC: 0.82	Occlusion Sensitivity	251 from OASIS-3
[68] 2022	AD	MRI T1w	MAXNet with dual attention module (DAM) and multi-resolution fusion module (MFM)	95.4%	High-resolution activation mapping (HAM), and a prediction-basis creation and retrieval (PCR)	ADNI—826 cognitively normal individuals and 422 Alzheimer’s patients
[90] 2022	Brain tumors (survival rate prediction)	MRI T1w, T1ce, T2w, FLAIR	CNN	71%	SHAP	235 patients from BraTS 2020
[91] 2022	Brain tumor	MRI	VGG16	-	SHAP	Kaggle
[69] 2022	AD: non-demented, very mild demented, mild demented and moderate demented	MRI	VGG16	78.12%	LRP	6400 images with 4 classes
[17] 2022	PD	Dopamine transporter (DAT) SPECT	CNN	95.8%	LRP	1296 clinical DAT-SPECT as “normal” or “reduced” from the PACS of the Department of Nuclear Medicine of the University Medical left Hamburg Eppendorf
[92] 2022	Psychosis	MRI	Neural network-based classifier	Above 72%	LRP	77 first-episode psychosis (FEP) patients, 58 clinical high-risk subjects with no later transition to psychosis (CHR_NT), 15 clinical high-risk subjects with later transition (CHR_T), and 44 HC from the early detection of psychosis project (FePsy) at the Department of Psychiatry, University of Basel, Switzerland
[70] 2021	AD vs. NC and pMCI vs. sMCI	MRI	Three-dimensional residual attention deep neural network (3D ResAttNet)	91% AD vs. NC, 82% pMCI vs. sMCI	Grad-CAM	1407 subjects from ADNI-1, ADNI-2 and ADNI-3 datasets
[93] 2021	PD	DAT SPECT	3D CNN	97.0%	LRP	1306 123I-FP-CIT-SPECT, PACS of the Department of Nuclear Medicine of the University Medical left Hamburg Eppendorf
[94] 2021	Age Prediction	MRI T1w	DNN	-	SHAP and LIME	ABIDE I—378 T1w MRI
[5] 2021	AD, MCI	EEG	SVM, ANN, CNN	Up to 96%	LIME	284 AD, 56 MCI, 100 HC
[95] 2021	Age estimation	Structural MRI (sMRI), susceptibility-weighted imaging (SWI) and diffusion MRI (dMRI)	DNN	-	SHAP and LIME	16394 subjects (7742 male and 8652 female) from UKB United Kingdom Biobank
[36] 2021	Brain tumor lower-grade gliomas and the most aggressive malignancy, glioblastoma (WHO grade IV)	MRI T2w	DenseNet121, GoogLeNet, MobileNet	DenseNet-121, GoogLeNet, MobileNet achieved an accuracy of 92.1, 87.3, and 88.9	Grad-CAM	TCGA dataset from The Cancer Imaging Archive repositories—354 subjects—19,200 and 14,800 slices of brain images with and without tumor lesions
[71] 2020	AD	MRI T1w	Variants of AlexNet, VGG16	-	Swap Test/Occlusion Test	ADNI Australian Imaging, Biomarker and Lifestyle Flagship Study of Ageing3 (AIBL)—training, validation, and test sets, each of them containing respectively 1779, 427, and 575 images

#### 3.2.3. Vision Transformers

Vision transformers (ViTs) [96], an alternative to CNN architectures designed for CV applications, decompose an input image into a series of patches and use self-attention mechanisms to model the relationships between these patches. This allows ViTs to capture long-range dependencies and global context more effectively, whereas CNNs require deep layers to build global understanding from local features. Compared to CNNs, ViTs are less data-efficient, but have a higher capacity and are computationally intensive. Some of the largest modern CV models are ViTs, including one with 22 billion parameters [97]. Hybrid architectures that possess properties of both CNNs and ViTs have also been observed in the literature employing them for XAI-powered CAD of neurological disorders. A few fo these studies have been summarized in Table 3.

#### 3.2.4. XAI-Powered CAD Using Multimodal Data

Few studies propose the use of multimodal data for the prediction and management of disorders. This offers extra diagnostic features which may support and enhance the accuracy of diagnoses. In [32], Jahan et al. use clinical, physiological, and MRI data for a five-class classification of AD using the OASIS dataset. They compare the performance of nine popular ML models and employ SHAP for explainability. Their study finds random forest (RF) to be the best-suited classifier for this job, with a 10-fold cross-validation accuracy of 98.81%. The research in [13] proposes concatenation of PET and MRI images for the diagnosis of AD using ResNet18. The authors claim that their explainable system has an accuracy of 73.90% using the ADNI dataset. In [99], Kamal et al. present the fusion of brain MRI with gene expression data for AD classification into four categories. They use CNN and LIME to achieve an accuracy of 97.6%. Similarly, in the study conducted in [100], the integration of 11 modalities, including PET, MRI, cognitive scores, genetic data, demographic data, patient history, CSF, neuropsychological battery, lab tests, etc., for the classification of AD can be observed. The use of multimodal data may certainly enhance the potential of accurate diagnoses by these CAD tools, yet no evaluation criteria of the explanations generated in these systems have been found. A summary of the recent XAI-powered CAD systems that employ multimodal data is given in Table 4.

#### 3.2.5. Evaluation of Clinical XAI

Some studies were found to focus on guidelines for the evaluation of clinical XAI in medical image analysis. Jin et al. [26] propose guidelines for choosing an XAI technique based on understandability, clinical relevance, truthfulness, informative plausibility, and computational efficiency. They implemented and evaluated 16 commonly used post-hoc heatmap XAI techniques, including gradient-based and perturbation-based techniques. Focusing on two tasks, i.e., (i) classifying gliomas as low-grade (LGG) and high-grade gliomas (HGG) using 3D CNN architecture and the BraTs 2020 dataset, and (ii) knee lesion identification, they concluded that all 16 XAI techniques were inadequate for clinical use due to their failure in truthfulness and informative plausibility. Another study in [30] proposes the modality-specific feature importance (MSFI) metric to evaluate feature attribution maps on multimodal medical images. The authors also highlight that the literature review indicates that 35% of studies evaluated the explanations with computational metrics only, 8% involved medical experts to verify explanation plausibility either quantitatively or qualitatively, and only a meager 5% employed both modes of evaluation.

#### 3.2.6. Medical Experts’ Feedback

Very few articles were found in the literature that incorporated medical experts’ feedback to assess their proposed systems’ clinical utility. The system proposed in [23] employs hybrid vision transformers and CNNs for glioma segmentation in brain MRI scans. They also claim to provide surgeon-understandable heatmaps to make their system transparent. In addition, they conducted structured interviews with two medical experts from the neurosurgery department at the University Hospital of Ulm to evaluate the practical utility of their developed system. Their discussions included an evaluation of the model’s performance with actual patient cases, and the interpretability of the model’s decision-making process with respect to the clinical experience of the neurosurgeons. According to the authors, the experts found Grad-CAM to be a valuable tool to introduce explainability/transparency into the otherwise opaque DL regimens. Another study in [101] employs a subtractive spatial lightweight CNN to classify malignant tumors as medulloblastomas, ependymomas, meningiomas, lymphomas, and anaplastic astrocytomas. They included CAM for explainability. After achieving a reasonable classification accuracy of up to 93.33% in the first evaluation phase, they took 10 doctors onboard to judge the meaningfulness of the generated explanations in the second phase. Each doctor went through a total of 120 MRI images and colored the tumor regions manually. These colorings were then compared with the heatmaps generated by CAM. According to the authors, the overlap never went below 98%, with it being 100% in the majority of the cases. They also conducted a survey among the doctors toward the end of their study, which included 11 statements such as “The system is trustworthy in terms of diagnosis of brain tumors”, “I am able to understand well about the detection by looking at the heatmaps”, “I am able to make decision faster thanks to XAI view of the system”, and “I want to use this system for auto-decision-making in brain tumor diagnosis”. These statements were rated by doctors on a scale of 1 to 5; with 5 indicating the strongest agreement. The average rating from all 10 doctors who participated in this study was above 4.5, with 4.8 being the highest. On the other hand, in our study, the doctors who were generous enough to find time for our survey regarding XAI in CAD were, unfortunately, mostly not generous at all in expressing their trust in XAI-powered CAD systems. Their opinions are presented in the subsequent section. We think that input from medical domain experts on XAI-powered CAD research is an absolute necessity, but it is severely lacking at the moment, as evident from the literature. The requirements of doctors—especially in terms of the modes of explanation (visual, textual, etc.),—must be identified in the first phase and incorporated into CAD tools in subsequent phases, followed by rigorous testing and validation by medical experts in clinical settings.

### 3.3. The Expert Opinion

**Figure 7:**
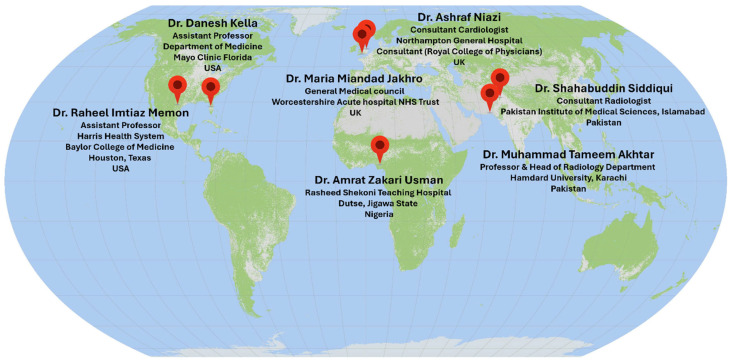
Details of the medical experts that participated in the survey on XAI-powered CAD tools for differential diagnosis of neurological disorders from brain MRI.

A survey was conducted among medical experts from all over the world; their opinions on the role of AI in CAD and the influence of the addition of “explainability” were recorded. The geographical locations and affiliations of the experts are given in Figure 7. Two questions were presented to the experts:

*1.*  *In your opinion, can AI be useful to act as a CAD tool for your assistance in reaching concrete diagnoses using medical imaging data?*
*2.*  *Would integration of “Explainability” to the AI (black box) models build your confidence and make you feel more comfortable in using such systems?*

A brief description of AI, its old-school black box architectures, and its role in CAD was provided for them to review. In addition, a general overview of XAI was also provided to them based on the research in [6], along with an example of brain tumor detection research in [21]. This included heatmaps generated by Grad-CAM technology in XAI, which highlight features in a brain MRI responsible for classifying the scan as a tumor. This was just to refresh their knowledge or introduce them to XAI in diagnostic radiology, as their past experiences with AI and XAI were unknown. Experts were chosen from different parts of the world, with different age groups and specializations, including neurology, radiology, and cardiology, to achieve a diverse set of opinions. On average, it took around 20 min for each of the experts to record their views, and their consent was obtained for publishing their views. The responses of the doctors are presented in this section to bring to light the exact feelings, expectations, and concerns of the actual end users of XAI-powered CAD systems.

**Expert 1—Dr. Danesh Kella**
*“Artificial intelligence, particularly machine learning algorithms, has shown significant promise in assisting healthcare professionals with the diagnosis of medical conditions using imaging data such as X-rays, MRIs, and CT scans. However, the routine integration of AI into medical imaging diagnosis is in its infancy, and several challenges and limitations exist. For instance, the interpretability of AI-generated diagnoses remains a significant concern, as clinicians need to understand the reasoning—the “black box”—behind the model’s recommendations to routinely incorporate it into clinical practice.*
*Yes, the explanation of the AI’s decision into AI models would likely increase confidence and comfort in using such systems. However, it would be even more helpful if the AI explains it in a manner akin to how a professor would explain to their trainees how a certain characteristic of the brain mass on MRI indicates the likelihood of a certain tumor. By providing human-like insights into how AI models arrive at their decisions, they offer transparency and clarity to users, including healthcare professionals.*
*Despite these challenges, AI has a definite role in the future of medicine; however, the extent and exact nature remain unclear. Collaborative efforts between AI programmers, healthcare professionals, and regulatory bodies are crucial to ensuring the safe and responsible deployment of AI technology in clinical settings.”*
**Expert 2—Dr. Amrat Zakari Usman**
*“No, I don’t think AI can be useful to act as a computer aided diagnosis, interpretation by clinician is important so that other differential diagnosis will be considered in the course of patients’ management.*
*No because I don’t think AI can give enough reason to back up a diagnosis.”*
**Expert 3—Dr. Raheel Imtiaz Memon**
*“While new and emerging technologies can certainly be an asset in the medical field, I have my reservations about the use of AI for diagnosis purposes. AI is still quite new and though efficient in many areas, it is not a perfect science. No AI can replicate time spent and experiences had during medical school, residency, and other post graduate training, or the value of peer consultation and appropriate bedside manner—all things which aid in appropriate diagnosis. While AI can be used as a tool in addition to those already being used, it should not be relied upon for diagnostic purposes.”*
**Experts 4, 5—Dr. Maria Miandad Jakhro & Dr. Ashraf Niazi**
*“As doctors, we believe that AI can be tremendously valuable as a Computer-Aided Diagnosis (CAD) tool in interpreting medical imaging data. The integration of AI into diagnostic processes has the potential to revolutionize how we approach patient care in terms of accuracy, efficiency, and accessibility. AI algorithms excel in analyzing vast amounts of medical imaging data with remarkable speed and precision. This capability can significantly aid healthcare professionals in interpreting complex images, identifying subtle abnormalities, and reaching accurate diagnoses. Moreover, AI-powered CAD systems can serve as a valuable second opinion, offering insights that complement and validate our own diagnostic assessments. One of the most compelling advantages of AI in medical imaging is its ability to enhance diagnostic efficiency. By rapidly processing imaging data, AI can expedite the diagnostic process, leading to quicker treatment decisions and improved patient outcomes, particularly in time-sensitive situations. Furthermore, AI has the potential to address challenges related to access to specialized expertise. In regions where there may be a shortage of experienced radiologists or specialists, AI-powered CAD tools can provide valuable support, ensuring that patients receive timely and accurate diagnoses regardless of geographic location. However, it’s important to recognize that AI should not replace the role of healthcare professionals. Rather, it should be viewed as a valuable tool that augments our diagnostic capabilities. As doctors, our clinical judgment, experience, and empathy are irreplaceable components of patient care that cannot be replicated by AI.*
*In conclusion, we believe that AI holds immense promise as a CAD tool in medical imaging, offering significant benefits in terms of accuracy, efficiency, and accessibility. Embracing AI technology alongside our expertise as healthcare professionals can lead to more effective diagnosis and treatment, ultimately enhancing the quality of care we provide to our patients.”*
**Expert 6—Prof. Muhammad Tameem Akhtar**
*“The set of diseases on which the AI is developed will be different in every part of the world with different sets of diseases with different clinical presentation and radiological pictures, unless a good number of histopathologically proven cases with images which are super imposed to reach a conclusive and definitive diagnosis. Hence, studies must be conducted in different parts of the world with the same set of these diagnosed diseases and the same quality of images to be as close to diagnosis as 95% accuracy.*
*The study must be multidisciplinary with multiple caste and religions as there are different pattern of disease in different foods, lifestyle patterns. It needs at least hundred thousand cases of similar characteristics, patterns to reach to consensus and accurate diagnosis in keeping in mind with multiple filters of age, sex, dietary habit living standards as well as environment.*
*Yes, AI has a definite role provided a large study must be performed with large number of patients. It cannot replace human integration and involvement can minimise errors. Diseases are classified as per ACR codes. It will take years to be confident about the accuracy of diagnosis and will take time to develop confidence and accuracy.*
*The images are self-exclamatory. There is discrepancy in actual area of involvement and adjacent tissue involvement. The extent of the disease is variable in different projections while the disease itself is limited with the effects widespread which may be due to changes in the chemistry in adjacent tissue; where multiple factors like compactness/looseness of cells, and chemistry of intra and extra cellular compartments, natural state and disease state of cells play a vital role. Again, the signals generated from the cells will vary according to human habitat, including diet, ethnicity, environment etc.”*
**Expert 7—Dr. Shahabuddin Siddiqui**
*“It is a good idea to develop a tool which can read images of the brain and diagnose certain diseases. If properly trained, I certainly believe it can delineate all the abnormal areas of the brain. It may be able to pick up the small lesions which may otherwise be missed by the human eye. This certainly will aid the Radiologists in making an accurate diagnosis of the patient. But practically, I don’t think this tool can itself establish the diagnosis of the patient. Most diseases do not follow typical imaging patterns. The signal characteristics and the location of the disease process can vary greatly from patient to patient. Similarly, many different neurological disorders can have overlapping imaging features. It will therefore require the background knowledge and experience in clinical neurology and radiology to differentiate the disease considering all the key factors including the patient’s epidemiology, clinical presentation (signs and symptoms), lab results and the imaging features, not just the imaging features alone. So all in all, I think it can help neurologists and radiologists with accurately picking up the imaging findings but establishing the accurate diagnosis of the patient per se will still be the job of the primary neurologist and radiologist.*
*The integration of explainability will certainly build my confidence and make me feel more comfortable using such systems. But I will always rely on my clinical and radiology expertise to make the diagnosis.”*

## 4. Discussion

This section provides an overall summary of the systematic review, includes expert opinions from healthcare and data science, and proposes a future course of action for harnessing the tremendous potential of AI and channeling it into diagnostic medicine for the benefit of society. We also present the challenges to be faced in this journey to bridge the gap between AI and routine clinical practice.

### 4.1. Crux of the Matter and the Way Forward

The experts from the healthcare sector, despite appreciating the potential of AI in diagnostic medicine, showed some concerns. Some seemed very opposed to AI, whereas others were not certain about the future role of AI in medicine. Most of them believe that while AI might never reach the perfection required in this highly sensitive field, it could serve as a reasonable source of quick second opinions. They seemed skeptical about routine integration of AI into medical imaging diagnosis due to the field still being in its infancy and having many limitations. Nevertheless, the majority believed that such CAD tools can work adequately if trained properly on very extensive datasets from histopathologically proven cases. The “black box” nature was also a concern for some of them, and they encouraged the integration of explainability into the inferences generated by these highly opaque DL models. They also provided amazing suggestions regarding the level of explainability that would be acceptable to them. Dr. Danesh suggested that “*It would be even more helpful if the AI explains it in a manner akin to how a professor would explain to their trainees*”. This could be a very interesting direction for data scientists, engineers, and programmers to work on; since medical experts are ultimately going to be the end users of XAI-powered CAD tools, their requirements should be the top priority for the developers of such systems.

On the other hand, completely opposing and stern comments from data scientists have also been observed in the literature. Zaharchuk et al. [102] feel that “*Deep learning has the potential to revolutionize entire industries, including medical imaging. Given the centrality of neuroimaging in the diagnosis and treatment of neurologic diseases, deep learning will likely affect neuroradiologists first and most profoundly. One concern is that if these approaches are successful, some work that radiologists have traditionally performed may become obsolete*”. Korfiatis and Erickson [103] present an even stronger statement: “*There has been a revolution in the world of computer science with new formulations of deep learning technology achieving performance that exceeds humans in the identification of content in images. This revolution has caused some to predict that computers might replace radiologists*”. Others [104,105,106,107,108,109] believe more in a ‘meet in the middle’ kind of approach, stating that “*Despite the limitations, the advantages of DL far outweigh its shortcomings, and thus, it will be an essential tool for diagnosis and prognosis in the era of precision medicine. Future research teams in medical imaging should integrate DL experts in addition to clinical scientists in their teams so as to fully harness the potential of DL.*” [110] and “*With the clearer pathogenesis of human brain disorders, the further development of deep learning techniques, and the larger size of open- source datasets, a human-machine collaboration for medical diagnosis and treatment will ultimately become a symbiosis in the future*” [111].

This presents a window of research opportunities for experts from both worlds to work together toward the design and development of XAI-powered CAD tools, as we believe that such a symbiosis is the only way forward. The combination of the experience and expertise of doctors and the ability of machines to examine the most intricate of details at pixel level—which the human eye might overlook—can greatly minimize the risks of invalid diagnoses, thereby saving precious lives.

### 4.2. The Challenges Ahead

Besides explainability, XAI offers many other advantages, including improved error analysis capabilities [6], verification of results, and prospects of model refinement [20]. Despite all that, it does not seem to be typically designed for clinical purposes [30]. This section sheds light on the limitations and current challenges standing between the field of XAI (and the CAD tools powered by it) and routine healthcare.

#### 4.2.1. Limited Training Datasets and Generalizability Issues

Datasets with limited labeling/annotations have been observed in most of the studies developing CAD tools for neurological disorders [14,28]. Most of the developed systems were found to employ training and testing data from a single online source. This results in generalizability issues, i.e., models trained in this way are very highly likely to fail when presented with data from unseen sources that were not used in training. The open sharing of anonymized neuroimaging data should therefore be encouraged and more public grand challenges should be introduced to prompt crowdsourcing [14] for solutions to problems.

#### 4.2.2. Current Focus Mostly on Optimizing Performance of CAD Tools

Currently, XAI seems to be in its infancy, and most of the energy and attention of researchers and data scientists is focused on the accuracy enhancement and performance optimization in CAD tools [100]. This might be one of the reasons for the current immaturity of XAI in CAD.

#### 4.2.3. Absence of Ground Truth Data for Explainability

At present, there are almost no annotated ground truth data for explainability, be it visual, textual, or in any other form. For example, for AD, several neuroimaging and clinical biomarkers labeled datasets can be found, but none exist that validate the heatmaps for AD generated by XAI algorithms [28]. This makes performance evaluation challenging for XAI systems [16].

#### 4.2.4. Focus on Single Modality

Most of the CAD research found in the literature is single modality-oriented. Very few multimodal studies were found. The same was found to be true for XAI-powered CAD tools. Correlations between interpretations of different modalities may contribute significantly to differential diagnosis [28] and thus demand attention. The CAD of neurological disorders is a tedious task, particularly in cases of neurodegenerative disorders like AD and PD, where no clearly evident findings are present on brain MRIs, as opposed to tumors, which can be seen vividly as an abnormal growth. Another set of such diseases is MS and NMO, for which differential diagnosis is extremely challenging even for medical experts, given the similarities in symptoms and lesion patterns on MRI scans [112]. MS and NMO are demyelinating diseases of the central nervous system (CNS) that produce lesions or plaques on the brain, spinal cord, and optic nerve [113]. Since the treatment and management of both diseases is different [114], and the treatment of MS might have adverse effects on NMO patients [115], early and accurate diagnosis is of paramount significance [116,117]. Studies show that 50–85% of cases involving these disorders show lesions on brain MRIs [53], which appear as hyper-intense regions on T2w and FLAIR scans [118]. In cases without brain lesions, analysis of spinal lesions and orbits can help [119]. In such cases, a CAD tool trained for differential diagnosis can perform significantly better if multimodal patient data are used for training before its deployment in real-time clinical settings.

#### 4.2.5. Are Only Visual Explanations Sufficient?

In this review, visual explanations were found to be the most dominant in recent research [25,29,30]. This is understandable, as medical imaging is primarily associated with visual tasks. But are they enough? Non-visual methods were observed to be hardly researched [25]. Some might not be content with only visual explanations, but be more interested in explanations akin to a professor teaching his trainees. Textual XAI approaches might bring the additional baggage of natural language processing (NLP) with them, but that is acceptable as long as our end users/healthcare professionals are satisfied.

#### 4.2.6. How to Judge XAI Performance?

Many researchers have proposed and developed XAI-powered tools, but very few have worked in the direction of their performance evaluation, either quantitative or qualitative [19,30]. The existing, scant engineer-centered performance evaluation paradigm needs to shift, and more involvement of medical experts (who are the ultimate users) needs to be ensured. There is an absolute need for the uniform adoption of standard assessment criteria for explainability across the research community [6].

#### 4.2.7. More Doctors Onboard, Please!

When it comes to XAI-powered CAD research, from data annotation, to training, to providing ground truth explanations, to qualitatively analyzing the results of such tools, nothing is materializable without medical experts’ involvement and contributions. Being the end users of such tools, their requirements need to be prioritized above all. The idea of the human (doctor) in the loop has been observed frequently in the literature [10], and has been termed as a prerequisite for the design, development, and use of XAI-based CAD applications [19].

#### 4.2.8. User Awareness

It is important to thoroughly explain the capabilities, advantages, and limitations of XAI in CAD research to users (patients and doctors) [19].

#### 4.2.9. Security, Safety, Legal, and Ethical Challenges

AI, along with all the explainability and interpretability associated with it, would face safety, legal, and ethical challenges, especially in the field of medicine [11]. Because of this field still being in its infancy [16], the regulations will also take time to mature.

Data security and privacy are pivotal in healthcare and diagnostic medicine, as patients’ personal information and medical records are involved [120]. With the increasing adaptation of digital healthcare solutions, concerns about security measures have also been increasing [121]. Various healthcare data security breaches have been reported in the literature. In 2020, an astounding 642 such cases were reported in the United States, with the unauthorized exposure of over 30 million healthcare records [122].

Digital health data, commonly referred to as the electronic health records (EHRs), play an important role in centralizing healthcare systems. By using these data, medical experts can have access to a patient’s entire medical history, including ailments and treatment regimens followed in the past. This information is vital for fast, accurate, and safe diagnosis, prognosis, and treatment [123]. These records are, however, highly confidential, since they contain the most private information about the patients [124]. Technologies such as cloud computing and remote access make these data vulnerable to cyber-attacks [125]. In the case of AI-based CAD regimens, the training data also comprise such medical information, including imaging data, patient history, and blood work. Among the many possible forms of cyberattacks on AI-powered CAD systems, ‘data poisoning’ is the most significant one [126]. Since an AI model makes decisions based on the data fed to it during training (at least in the case of supervised learning), any change in those data can result in abnormal inferences. Optimistically thinking, this cannot cause physical harm to the patient, but in a worst-case scenario, serious damage can be caused by choosing a wrong treatment path due to an inaccurate diagnosis. XAI can help curb such issues, where a doctor can identify the invalid explanations generated by the CAD tool, reject the diagnosis, and request a technical inspection and reevaluation of the underlying AI regimen. This would not be possible with old-school ‘black box’ AI, where an inference is supposed to be blindly trusted. On the other hand, in the case of smart medical devices, cyber-attacks can result in catastrophic consequences; for example, imagine an insulin pump under such an attack [126]. Given the increasing severity of such cyber threats with advancements in technology [124], stern security measures are reuired, including encryption protocols, patient data anonymization, access control protocols, and XAI, along with periodic security audits [127].

#### 4.2.10. Let There Be Symbiosis!

It is important for doctors and data scientists to work together in this direction. The expertise of radiologists in identifying abnormalities from medical imaging data and that of engineers in developing software are equally important to accomplish this task. Hence, a symbiosis seems to be the only way forward. In addition, specialized training combining mathematics, data science, and medicine can be imparted to expedite research in this direction [6]. Nevertheless, it seems quite premature to comment on how long it would take to reduce the gap between the medical and AI domains to zero.

## 5. Conclusions

Interpretability and explainability, although absolute necessities for AI, especially in diagnostic medicine, still have a long way to go to achieve the required levels of maturity for their integration into regular medical practice. In addition to the lack of generalizability in AI-powered CAD tools, the scarcity of histopathologically proven and labeled datasets is another major shortcoming faced by currently available CAD tools. The key takeaways from this study can be summarized as follows:

1. The integration of explainability in such CAD tools will surely increase the confidence of medical experts, but the current modes of explanation might not be enough. More thorough and human professor-like explanations are what the healthcare professionals are looking for.
2. The quantitative and qualitative evaluation of such XAI schemes requires a lot of attention. The absence of ground truth data for explainability is another major concern at the moment which needs special attention, along with legal, ethical, safety, and security issues.
3. None of this can be achieved without uniting both medical professionals and scientists to work toward this cause in absolute symbiosis.

Failing to harness and channel the tremendous recent advances in technology, computational resources, and AI into healthcare would be an ultimate waste when they have the potential to improve the quality of life of those suffering from neurodegenerative disorders. It is extremely comforting to imagine a world with reduced mortality where fast, accurate, and reliable second opinions from XAI-powered CAD tools are embedded within healthcare systems, helping doctors to avoid delays and errors in differential diagnoses, with the result of saving precious lives.

## Figures and Tables

**Table 1:** XAI CAD research for neurological disorders using ML techniques. The table contains the year of study, the pathology diagnosed, the modality used, the AI technology employed, the accuracy of the proposed system, the XAI technology embedded, and the dataset used for training the systems.

Study	Pathology	Modality	Technology	Accuracy	XAI	Dataset
[65] 2024	Glioma	23 Clinical and Molecular/mutation factors	RF, decision trees (DT), logistic regression (LR), K-nearest neighbors (KNN), Adaboost, Support Vector Machine (SVM), Catboost, Light Gradient-Boosting Machine (LGBM) classifier, Xgboost, CNN	88% for Xgboost	SHAP, Eli5, LIME, and QLattice	Glioma Grading Clinical and Mutation Features Dataset—352 Glioblastoma Multiforme (GBM), 487 LGG patients
[32] 2023	AD, cognitively normal, non-Alzheimer’s dementia, uncertain dementia, and others	Clinical, Psychological, and MRI segmentation data	RF, LR, DT, MLP, KNN, GB, AdaB, SVM, and Naïve Bayes (NB)	98.81%	SHAP	OASIS-3, ADRC clinical data, Number of NC, AD, Other dementia/Non-AD, Uncertain, and Others are 4476, 1058, 142, 505, and 43, respectively
[21] 2023	Brain tumor	MRI	DenseNet201, iterative neighborhood component (INCA) feature selector, SVM	98.65% and 99.97%, for Datasets I and II	Grad-CAM	Four-class Kaggle brain tumor dataset and the three-class Figshare brain tumor dataset
[4] 2022	AD, EMCI, MCI, LMCI	MRI T1w	DT, LGBM, LR, RF and Support Vector Classifier (SVC)	-	SHAP	ADNI3—475 subjects, including 300 controls (HC, 254 Cognitively Normal and 46 Significant Memory Concern) and 175 patients with dementia (comprising 70 early MCI, 55 MCI, 34 Late MCI and 16 AD)
[66] 2020	AD	T1w volumetric 3D sagittal magnetization prepared rapid gradient-echo (MPRAGE) scans	DT and RF	Average 91%	Argumentation-based reasoning frame-work	ADNI—NC 144 and AD 69

**Table 3:** XAI CAD research for neurological disorders using vision transformers. The table contains the year of study, the pathology diagnosed, the modality used, the AI technology employed, the accuracy of the proposed system, the XAI technology embedded, and the dataset used for training the systems.

Study	Pathology	Modality	Technology	Accuracy	XAI	Dataset
[98] 2024	AD	MRI	Transfer learning (TL), vision transformer (ViT)	TL 58%, TL ViT Ensemble 96%	-	ADNI
[23] 2024	Gliomas segmentation	Three-dimensional pre-operative multimodal MRI scans including T1w, T1Gd, T2w, and FLAIR	Hybrid vision transformers and CNNs	Dice up to 0.88	Grad-CAM—TransXAI, post-hoc surgeon understandable heatmaps	BraTS 2019 challenge dataset including 335 training and 125 validation subjects

**Table 4:** XAI CAD research for neurological disorders using multimodal data. The table contains the year of study, the pathology diagnosed, the modality used, the AI technology employed, the accuracy of the proposed system, the XAI technology embedded, and the dataset used for training the systems.

Study	Pathology	Modality	Technology	Accuracy	XAI	Dataset
[13] 2023	AD	PET and MRI	Modified Resnet18	73.90%	-	ADNI—412 MRIs and 412 PETs
[99] 2021	AD	MRI and gene expression data	CNN, KNN, SVC, Xboost	97.6%	LIME	Kaggle—6400 MRI images, gene from the dataset OASIS −3, NCBI database, which contains 104 gene expression data from patients
[100] 2021	AD, MCI	11 modalities—PET, MRI, Cognitive scores, Genetic, CSF, Lab tests data, etc.	RF	93.95% for AD detection and 87.08% for progression prediction	SHAP—these explanations are represented in natural language form to help physicians understand the predictions	ADNI—294 cognitively normal, 254 stable MCI, 232 progressive MCI, and 268 AD

## Data Availability

No new data were created or analyzed in this study. Data sharing is not applicable to this article.
